# Effects of epiplakin-knockdown in cultured corneal epithelial cells

**DOI:** 10.1186/s13104-016-2082-7

**Published:** 2016-05-20

**Authors:** Masahide Kokado, Yuka Okada, Takeshi Miyamoto, Osamu Yamanaka, Shizuya Saika

**Affiliations:** Department of Ophthalmology, Wakayama Medical University School of Medicine, 811-1 Kimiidera, Wakayama, 641-0012 Japan

**Keywords:** Epiplakin, Knockdown, Corneal epithelium, Culture, Migration, Proliferation, E-cadherin

## Abstract

**Background:**

To investigate effects of knockdown of epiplakin gene expression on the homeostasis of cultured corneal epithelial cell line. We previously reported acceleration of corneal epithelial wound healing in an epiplakin-null mouse.

**Methods:**

Gene expression of epiplakin was knockdowned by employing siRNA transfection in SV40-immortalized human corneal epithelial cell line. Protein expression of E-cadherin, keratin 6 and vimentin was examined by western blotting. Cell migration and proliferation were examined by using scratch assay and Alamar blue assay, respectively.

**Results:**

Scratch assay and Alamar blue assay showed migration and proliferation of the cells was accelerated by epiplakin knockdown. siRNA-knockdown of epiplakin suppressed protein expression of E-cadherin, keratin 6 and vimentin.

**Conclusions:**

Decreased expression of E-cadherin, keratin 6 and vimentin might be included in the mechanisms of cell migration acceleration in the absence of epiplakin. The mechanism of cell proliferation stimulation by epiplakin knockdown is to be investigated.

## Background

Epiplakin was one of intermediate filament-related components and was originally identified as an autoantigen that reacted with serum from a patient with subepidermal blistering disease [1], [2]. Epiplakin is homologous to plectin and other members of the plakin family [1], [2]. Human epiplakin is a 552-kDa protein that is expressed in various epithelial tissues, i.e., epidermis, esophagus, outer root sheath of hair follicles and mucous epithelial cells [1], [2]. Studies by using an epiplakin-null mouse line showed that lacking epiplakin accelerates migration of epidermal keratinocytes in mice in vivo and also showed that this is also the case in outgrowth of keratinocytes from explanted skin tissue in vitro, although the exact mechanism of the phenomena is to be revealed [3]. We reported that corneal epithelium, a non-keratinizing stratified squamous epithelium, also express epiplakin and its loss accelerates cell migration-dependent healing of the corneal epithelium in mice. The in vivo study showed reduced E-cadherin protein and mRNA in epiplakin -null corneal epithelium, although E-cadherin expression level is not altered in epidermis by the loss of epiplakin [4]. In the present study we investigated behaviors, i.e., cell migration and proliferation, of epiplakin-knockdowned cultured corneal epithelial cell line and also examined the expression level of E-cadherin, keratin 6 and vimentin, both members of intermediate filament involved in cell migration [5], in the cells, although decreased epiplakin expression does not solely explain the mechanism of cell migration acceleration in corneal epithelium.

## Methods

Experimental protocols and the use of experimental mice were approved by the DNA Recombination Experiment Committee and the Animal Care and Use Committee of Wakayama Medical University and conducted in accordance with the Association for Research in Vision and Ophthalmology Statement for the Use of Animals in Ophthalmic and Vision Research.

Araki-Sasaki SV40-immortalized human corneal epithelial cell line was obtained from RIKEN Laboratories, Tsukuba Japan. The phenotype of this cell line retains many of the properties identified in their primary cell counterpart [6]. They were grown to confluence in DMEM/F12 medium containing 200 U/ml of penicillin and streptomycin, 5 % FBS, 0.1 μg/ml cholera toxin, 5 μg/ml insulin, and 10 ng/ml human epidermal growth factor (EGF).

## Knockdown of epiplakin in a corneal epithelial cell line in vitro

Expression of epiplakin was knockdowned in Araki-Sasaki cell line by employing a commercially available siRNA for epiplakin. The cells (2 × 10^5^) in each well of a six well plate in 2 ml antibiotic-free normal growth medium supplemented with 5 % fetal bovine serum. The cells were incubate at 37 °C under 5.0 % CO_2_ until the cells are 60–80 % confluent. Human epiplakin 1 siRNA (sc-77799, Santa Cruz Biotechnology, CA) or control siRNA (sc-37007, Santa Cruz Biotechnology) was transfected for 6.0 h according to the protocol prepared by the manufacturer using siRNA Transfection Medium (sc-36868, Santa Cruz Biotechnology). Cells without siRNA were also prepared (control). Normal growth medium (1.0 ml) containing 2 times the normal serum and antibiotics concentration (2× normal growth medium) was added to each well without removing the transfection mixture.

## Cell migration by scratch assay

The cells were cultured for 72 h after siRNA transfection procedure. The medium was then replaced with fresh 1× normal growth medium and processed for scratch assay as previously reported. Two liner defects were produced by a silicone needle in monolayer of the cells in control culture and siRNA epiplakin knockdown culture. Six cultures were prepared for each condition. The closure of the defect was evaluated at two independent points in each defect under phase-contrast microscopy at every 3 h until 30 h post-wounding. The width of the remaining defect was measured at four independent points at each time point and statistically analysed by Mann–Whitney U-test.

## Cell proliferation assay

Araki-Sasaki cells were treated with epiplakin-siRNA or control siRNA as described above. Cells were seeded into wells of 96-well plates at the concentration of 1.0 ×10^4^ cells/100 l/well 72 h after siRNA treatment, and incubated for 24 h. Twenty-six wells were prepared for each condition. Cell proliferation was assayed by using Alamar blue (Trek Diagnostic Systems, West Sussex, UK) according to the manufacturer’s protocol [7]. After a wash with phosphate-buffered saline (PBS), 40 μL Alamar blue was diluted in culture medium (1:2).Thirty and sixty min later, the optical absorbance at 570 nm was measured at 570 nm.

## Western blotting for epiplakin, E-cadherin, keratin 6 and vimentin

The efficacy of knockdown of epiplakin protein expression was first evaluated by using western blotting with anti-epiplakin antibody (T-16, sc-87104, Santa Cruz:diluted 1:500) as previously reported [8]. Then protein expression of E-cadherin, keratin 6 and vimentin was assayed by western blotting as previously reported [8]. SDS-PAGE was done with the gel of Mini-PROTEAN^®^ TGX™ Gel (Bio-Rad, #456-1096) under 200 V. The PVDF membrane was Immobilon-P Membrane, PVDF, 0.45 µm, 26 × 26 cm sheet.

(Merk Millipore Corporation, #IPVH304F0) and transfer the proteins under 5 V. Antibodies used were anti-E-cadherin antibody (G-10, sc-8426, Santa Cruz Biotechnology, diluted 1:1000) and anti-kerarin 6 antibody (LHK6, sc-53260, Santa Cruz Biotechnology, diluted 1:1000), and anti-vimentin antibody (C-20, sc-7557, Santa Cruz Biotechnology, diluted 1:1000).

## Results and discussion

**Fig. 1:**
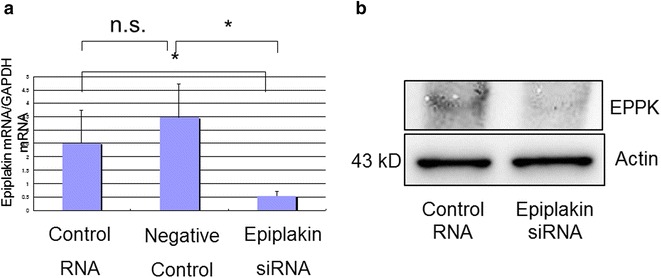
Epiplakin knockdown in cultured corneal epithelial cell line. Epiplakin knockdown by siRNA protocol nicely suppressed expression of epiplakin mRNA (**a**) and protein (**b**) as compared with control cells with control siRNA transfection

**Fig. 2:**
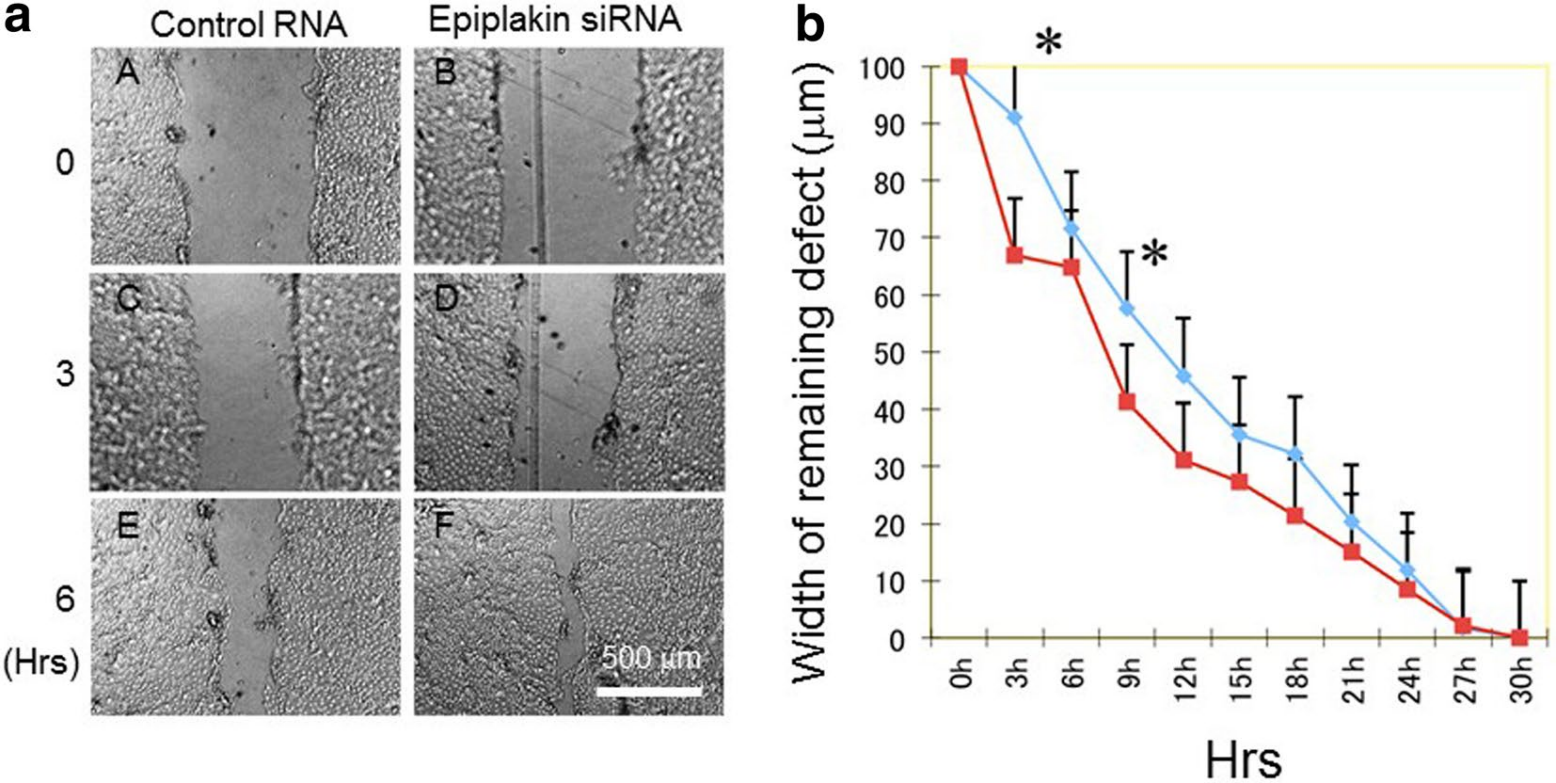
Effects of knockdown of epiplakin in cell migration. A liner defect was produced in confluent culture of control and epiplakin-knockdowned cells and was allowed to closed by cell migration. The width of the remaining defect was significantly smaller in epiplakin siRNA test culture (*red*) as compared with control siRNA culture (*blue*) at 3 and 9 h post-scratching (**a**). *Bar* 500 m. Frame **b** shows the time course of defect closure in cell culture either with epiplakin siRNA or control siRNA

**Fig. 3:**
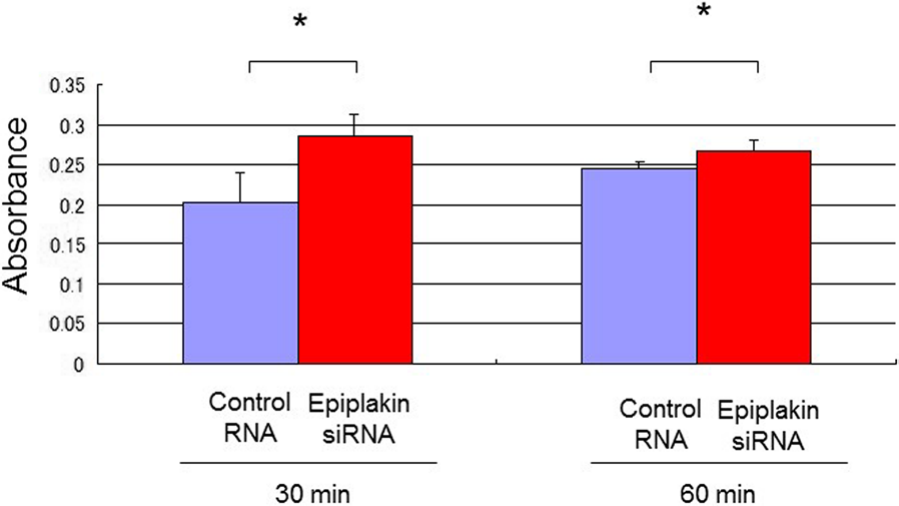
Effect of epiplakin knockdown on cell proliferation. Control and epiplakin-knockdowned cells were grown in wells of 96-well plates and Alamar blue dye was supplemented the culture medium. Relative absorbance of Alamar blue dye reaction in the medium was monitored to examine the activity of cell proliferation. Epiplakin-siRNA knockdown significantly promoted cell proliferation as compared with control siRNA culture at 30 and 60 min (Fig. 3). Evaluated as 95 % significant with the p value of 1.07032763506866 × 10 (−13) (< 0.101,076) in the datum of 30 min, and p value = 1.87138814984332 × 10 (−8) (p < 0.028528) in the datum of 60 min

Present study was undertaken to uncover the roles of epiplakin in modulation of behaviors of cultured corneal epithelial cell line. Our previous in vivo study revealed that deletion of epiplakin gene in a mouse suppressed cell proliferation but accelerated migration-dependent wound healing in corneal epithelium in association with reduction of E-cadherin expression, although the mechanism was to be explored [4]. In the current study we employed siRNA knockdown for this purpose. Epiplakin siRNA nicely suppressed expression of epiplakin at the mRNA and protein levels as compared with cells with control siRNA transfection (Fig. 1). In scratch assay the time course of defect closure in cell culture indicated that epiplakin siRNA accelerated wound closure. As shown in Fig. 2, the width of the remaining defect was significantly smaller in epiplakin siRNA test culture as compared with control siRNA culture at 3 and 9 h post-scratching. The in vitro datum coincides our previous finding of cell migration promotion in corneal epithelium of a epiplakin-null mouse. We and others reports that p38 signal or c-Jun-*N*-terminal kinase modulates cell migration of corneal epithelium [9–11]. It is to be clarified whether the loss of epiplakin affects directly the signaling cascades that involves migration of alteration of cytoskeletal structures might affect cell movement. On the other hand, Alamar blue assay showed that epiplakin siRNA knockdown significantly promoted cell proliferation as compared with control siRNA culture at 30 and 60 min (Fig. 3). Cell proliferation in in vivo corneal epithelium was suppressed by the loss of epiplakin. The reason for this discrepancy is to be uncovered. Cell–cell contact molecules such as Wnt/b-catenin is reportedly involved in cell proliferation modulation [12]. It is to be examined if cell culture condition may affect cell adhesion mechanism related to cell proliferation.

**Fig. 4:**
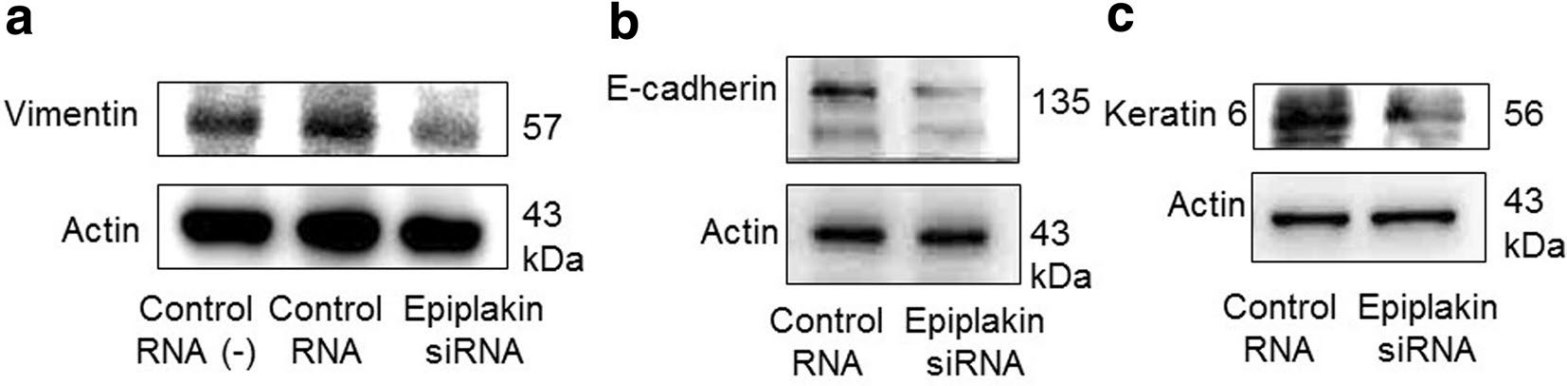
Effect of epiplakin knockdown on protein expression of vimentin, E-cadherin and keratin 6. Control and epiplakin-knockdowned cells were grown in wells of 60-min culture dishes and the cells were collected for western blotting for vimentin, E-cadherin and keratin 6 as described in the manuscript. Expression of vimentin (**a**), E-cadherin (**b**) and keratin 6 (**c**) was suppressed by siRNA knockdown of epiplakin as compared with their expression level in cells with control siRNA transfection as detected by using western blotting

Expression of each of E-cadherin, keratin 6 or vimentin was suppressed by siRNA knockdown of epiplakin as compared with expression level of these components in the cells with control siRNA as detected by using western blotting (Fig. 4). There is a possibility that suppression of expression of these three components might be included in the mechanism of promotion of migration of corneal epithelial cells. Decreased expression of E-cadherin was also observed in in vivo corneal epithelium of an epiplakin-null mouse [4]. A cell culture experiment previously showed that expression of a desmosome-related component affects E-cadherin expression; overexpression of desmoglein-3 decreases E-cadherin expression [13]. The loss of E-cadherin reportedly promotes the conversion of an epithelial cell to that of more migratory phenotype [14, 15]. Investigations by using mutant mice suggest that desmosomal components perform modulating effects on cell proliferation (positively or negatively) or differentiation. For example, keratinocyte proliferation initiated through downregulation of desmoplakin by RNA interference in cell culture [16]. This might be also the case in in vivo condition; mice lacking desmocolin-1 exhibited epidermal hyperplasia, in association with suprabasal keratinocyte proliferation and alteration of expression pattern of other desmosomal components [17]. Overexpression of desmoglein-2 or -3 induces keratinocyte hyperproliferation in the epidermis suggesting that this desmoglein-2/3 positively regulate cell cycle progression [18, 19]. Plakoglobin participates in the regulation of the c-Myc gene and highlights the importance and additional function of desmosomal constituent proteins in the control of cell proliferation [20]. However, molecular mechanisms of modulation of cell proliferation in association with an alteration of expression level of a desmosomal component is still puzzling. Keratin 6 is reportedly involved in migration of epithelial cell types including keratinocytes [3]. Decreased expression of this intermediate filament component might also be included the mechanism of migration acceleration of corneal epithelial cells in vitro. In the current in vitro experiment expression of vimentin was also suppressed by epiplakin-knockdown. Although vimentin-intermediate filament is also reportedly involved in cell migration [21], here decreased expression of vimentin was associated with cell migration promotion. The cell biology underlying this phenomenon is also to be investigated.

Further study is to be conducted to uncover the mechanism of cell migration acceleration in the absence of epiplakin in vivo and in vitro in order to understand the biological mechanism underlying corneal epithelial wound healing.

## Conclusions

SiRNA knockdown of epiplakin gene expression accelerated migration and proliferation of corneal epithelial cell in cell culture. Decreased expression of E-cadherin, keratin 6 and vimentin might be included in the mechanisms of cell migration acceleration in the absence of epiplakin. The mechanism of cell proliferation stimulation by epiplakin knockdown is to be investigated.
